# South Indian Cuisine with Low Glycemic Index Ingredients Reduces Cardiovascular Risk Factors in Subjects with Type 2 Diabetes

**DOI:** 10.3390/ijerph17176232

**Published:** 2020-08-27

**Authors:** Nivedita Pavithran, Harish Kumar, Arun Somasekharan Menon, Gopala Krishna Pillai, Karimassery Ramaiyer Sundaram, Omorogieva Ojo

**Affiliations:** 1 Department of Clinical Nutrition, Amrita Institute of Medical Sciences and Research Centre, Amrita Vishwa Vidyapeetham, Kochi, Kerala 682041, India; brinivedita@aims.amrita.edu; 2 Department of Endocrinology, Amrita Institute of Medical Sciences and Research Centre, Amrita Vishwa Vidyapeetham, Kochi, Kerala 682041, India; harishkumar@aims.amrita.edu (H.K.); or arunsmenon@aims.amrita.edu (A.S.M.); 3 Department of General Medicine, Amrita Institute of Medical Sciences and Research Centre, Amrita Vishwa Vidyapeetham, Kochi, Kerala 682041, India; mgkpillai@aims.amrita.edu; 4 Department of Biostatistics, Amrita Institute of Medical Sciences and Research Centre, Amrita Vishwa Vidyapeetham, Kochi, Kerala 682041, India; krsundaram@aims.amrita.edu; 5 School of Health Sciences, Faculty of Education, Health and Human Sciences, University of Greenwich, London SE9 2UG, UK

**Keywords:** low GI diet, south Indian (Kerala) cuisine, hs-CRP, HOMA-IR, ApoB, glycemic control

## Abstract

*Background*: Inflammation is considered as a predictor of cardiovascular diseases in type 2 diabetes mellitus. No previous studies have investigated the effect of low glycemic index (LGI) recipes of South Indian cuisine on the risk factors of cardiovascular disease in patients with diabetes. *Aim*: The aim of this randomized controlled trial was to evaluate the improvement in cardiovascular risk factors and blood glucose control, in patients with type 2 diabetes, after intervention with recipes of Kerala cuisine, from locally available whole grain cereals, low in glycemic index. *Method*: This was a prospective and randomized controlled study that was conducted over a period of 24 weeks. A total of 80 participants were recruited from the Department of Endocrinology and Diabetes Outpatient in Kerala, South India. All 80 patients had type 2 diabetes, and were aged between 35 and 65 years. Participants were randomly assigned and advised to follow either a LGI diet plan (*n* = 40) or their usual diet, which served as a control group (*n* = 40). The advice was reinforced throughout the study period. Anthropometric, biochemical parameters which included glycemic and cardio-metabolic parameters were measured according to standard procedures. T-tests were conducted to compare the differences between intervention and control groups, and the Pearson correlation coefficient was used to evaluate associations between the variables. *Results*: There were significant differences (*p* < 0.05) between the intervention and control groups with respect to weight, HbA1c, insulin, triglycerides, Homeostatic Model Assessment of Insulin Resistance (HOMA-IR), high sensitivity C-reactive protein (hs-CRP) and apolipoprotein B (ApoB). There was also a positive correlation between weight and blood glucose variables. ApoB was positively correlated with lipid profile and insulin levels. *Conclusions*: The long-term implementation of LGI diet of Kerala cuisine has been found to promote weight loss, enhance insulin sensitivity and reduce the cardiovascular risk.

## 1. Introduction

Diabetes mellitus is a well-established risk factor for cardiovascular disease, and an increased incidence of coronary artery disease and cardiovascular mortality has been strongly associated with the presence of metabolic syndrome [1] This linkage has been corroborated by the notably higher morbidity and mortality rate in patients with diabetes compared to non-diabetic individuals. Besides, metabolic syndrome has been reported to be a predictor of subclinical atherosclerosis. Some of the factors associated with the increased risks of cardiovascular disease in diabetes mellitus are higher levels of low density lipoprotein (LDL) cholesterol, elevated blood pressure, lower levels of high density lipoprotein (HDL) cholesterol, insulin resistance, hyperglycemia, inflammation, hypertension, dyslipidemia and obesity [2]. Vascular dysfunction resulting from type 2 diabetes has been shown as the causative factor for cardiovascular disease [3].

Inflammatory-response proteins are considered as predictors of cardiovascular events. C-reactive protein (CRP) is defined as a sensitive, but non-specific marker of inflammation [4]. The atherosclerotic process is now considered as an inflammatory disease. Compared to other ethnic groups, South Asian community is highly vulnerable to higher mortality rates from coronary heart disease (CHD) and stroke. Among Asians, incidence of CHD, 5–10 years earlier, have been ascribed to lower HDL, higher triglyceride and higher LDL levels and higher blood pressure (BP). Furthermore, a strong correlation has been found between diabetes and higher rates of heart failure (HF) and all-cause death (ACD) [5]. Higher incidences of cardiovascular (CVD) complications in the Asian population with type 2 diabetes mellitus (T2DM) calls for systematic treatment strategies tailored to Asian ethnicity [6].

Cereals are the staple diet of South Indians and carbohydrate constitutes about 55–65% of their daily intake of calories. There has been a transition in the quality of grains consumed from whole grains to highly polished grains. Consumption of whole grains is highly recommended unlike refined grains, which comprise endosperm that are known to stimulate adverse effect on the cardio-metabolic risk factors including diabetes [7]. Ingestion of high carbohydrate diets can raise blood glucose, insulin and triglycerides, leading to insulin resistance. Glycemic index (GI), which gauge the quality of carbohydrates, is considered as an important factor in the management of non-communicable diseases, particularly type 2 diabetes. Glycemic index is a relative ranking of carbohydrate in foods according to how they affect blood glucose levels. Carbohydrates with a low GI value (55 or less) are more slowly digested, absorbed and metabolized and cause a lower and slower rise in blood glucose and, therefore, insulin levels. High glycemic index foods(>70) have been shown to trigger rapid increase in the post-prandial blood sugar and insulin [8] and have shown to increase the risk of type 2 diabetes mellitus and CVD [9,10] in the western population as well as in Asian population, specifically Indians [11] and Chinese [12].

Traditional Indian diets are rich in dietary fiber, which could explain the lower incidence of type 2 diabetes mellitus in India in the 1960s–1970s. Studies in western countries have shown that the replacement of refined grains with whole grains have resulted in risk mitigation of type 2 diabetes and CVD in hyperinsulinemic and impaired glucose tolerant patients.

In our previous study [13] that investigated the effect of a low GI (LGI) diet with local recipes of South Indian, Kerala cuisine on body composition and glycemic control in people with diabetes, it was demonstrated that there was a significant reduction (*p* < 0.05) of truncal obesity and glycated hemoglobin. However, there is minimal published testimony that shows the efficacy of long-term intervention, triggered by smaller changes in the intake of traditional red rice and whole wheat flour that is low in glycemic index for the risk reduction of CVD in South Indians with type 2 diabetes. The current study is a follow up component of a larger study directed to assess the improvement in cardiovascular risk factors and blood glucose control among type 2 diabetes subjects, pursuant to a 24-week dietary consumption of Kerala cuisine from locally available LGI whole grain cereals.

### 1.1. Objectives of this study:

#### *Primary Objective* 

- To compare the reductions in glycated hemoglobin (HbA1c) after 24 weeks of intervention with LGI diet

### 1.2. Secondary Objectives

- To examine changes in blood lipids and blood pressure in the intervention group on LGI diet, compared to the regular diet.
- To evaluate changes in inflammatory markers such as hs-CRP and apolipoprotein B (ApoB) between the LGI and control group.
- To measure reduction in fasting insulin and Homeostatic Model Assessment of Insulin Resistance (HOMA-IR) in the study groups, after intervention with the diet.
- To observe effects of LGI diet in reducing obesity and abdominal obesity
- To correlate weight changes and cardiovascular risk factors in subjects with type 2 diabetes

## 2. Methodology

The study was a single-centered, controlled trial, where randomized sampling technique was used to recruit the subjects. Every third patient with type 2 diabetes who visited the Dietitian in the Department of Endocrinology and Diabetes (OP), Amrita institute of Medical Sciences and Research Centre, Kerala, India was screened and assigned at random to either the interventional or the control group. A total of 120 patients were randomized into either LGI group or control group.

*Study duration:* The study was conducted between October 2018 and April 2019 for a period of 24-weeks. 

*Inclusion criteria:* Subjects with history of type 2 diabetes aged between 35 and 65 years, of both genders with HbA1c in the range of 7 to 10%, who were managed by stable doses of antidiabetic medications for at least 3 months prior to enrolment, and had no history of CVD, were included in the study. Patients on antihypertensive and/or cholesterol lowering medications were also managed by stable doses of these medications for at least 3 months prior to enrolment. All medications remained unchanged during the whole study. 

*Exclusion criteria:* Subjects with chronic diabetes >10 years; other diabetes associated complications such as diabetic retinopathy, nephropathy, neuropathy; conditions like cancer, chronic renal failure; chronic cardiovascular ailments such as chronic cardiac failure or subjects having triple vessel disease or double vessel disease requiring surgical management; subjects who have undergone major surgeries and conditions like psychiatric illness or malabsorption syndromes were excluded from the study group. After screening, informed consent was obtained from all the study participants. The study was approved by the institutional ethics committee.

### 2.1. Sample Size

A pilot study was conducted to estimate the minimum sample size. Since there was no published paper in the existing literature covering foods of Kerala cuisine and cardiovascular risk factors in type 2 diabetes, a pilot study was performed with 20 subjects each in the LGI diet group and the control group. Based on the primary objective variable, HbA1c(%), the mean difference in LGI group was −1.13 ± 0.27 and control group was 0.4 ± 0.25, with 80% power and 95% confidence, and the minimum sample size was found to be 16 in each group. Among a total of 120 subjects with type 2 diabetes, only 80 participants completed the study, 40 in each group. This sample size will yield >90% power and 99% confidence for the comparison.

### 2.2. Study Procedures

Anthropometric measurements which included height, weight, BMI, waist circumference, hip circumference and blood samples were taken at baseline and after 6 months. Height was measured without shoes, to the nearest 0.1 cm, using a stadiometer; weight was measured by a calibrated weighing scale to the nearest 0.1 kg. BMI was calculated and interpreted according to the Asian classification: normal (18.5–22.9 kg/m^2^), overweight (23.0–24.9 kg/m^2^), Obese class I (25.0–29.9 kg/m^2^) and Obese class II (>30.0 kg/m^2^) [14]. 

Waist circumference was measured to the nearest 0.5 cm between the iliac crest and the lowest rib at the narrowest point just above the hip bone; hip circumference was measured around the maximum circumference of the buttocks using an inch tape. 

Blood pressure was measured with the subject in the seated position using a sphygmomanometer; a consistent value above 130/80 mmHg was considered to be hypertensive according to the New ACC/AHA high blood pressure guidelines (2019) [15].

### 2.3. Biochemical Analysis

A blood sample for fasting blood glucose was collected after 8 to 12 h of fasting in a grey vacutainer containing an anticoagulant and a stabilizer, i.e., EDTA, and sodium fluoride and postprandial blood glucose samples were collected in another grey vacutainer 2 h after meal. Whole blood was collected in a violet vacutainer coated with EDTA K2 for the estimation of glycated hemoglobin (HbA1c). 

The blood sample for analyzing fasting lipid profiles, fasting insulin and ApoB were collected using green vacutainer coated with lithium heparin, ammonium heparin or sodium heparin. 

HOMA-IR was calculated as fasting insulin (U/L) × fasting glucose (mg/dL)/405, as described by Matthews et al. [16]. 

The blood samples were spun immediately after sampling; the plasma collected, refrigerated and stored at −20 °C until all the patients completed the study. All samples were estimated using the Roche autoanalyzer, at the central laboratory of Amrita Institute of Medical Sciences and Research Centre, tertiary care hospital accredited by the National Accreditation Board of Testing and Calibration Laboratories (NABL). The interpretation of the blood glucose profile was assessed according to American Diabetes Association (ADA) guidelines 2017, and the reference interval for lipids was appraised per National Cholesterol Education Program (NCEP) Adult Treatment Panel III Report [17].

### 2.4. Dietary Intervention

The dietary assessment was done in all the study subjects. The quality and quantity of the consumed diet was assessed by using food frequency questionnaire and 24-h dietary recall method, respectively. Macronutrient composition of the consumed diet was calculated from the 24-h dietary recall using Nutritive value of Indian Foods. The Food Frequency Questionnaire (FFQ) included 60 food items commonly consumed in Kerala. The frequency of food intake was recorded as either daily, weekly, monthly, occasionally and never. The compliance to LGI ingredients in the intervention group was assessed from the FFQ when the subjects came for follow up.

The control group was advised to consume their usual diet. The LGI group was advised to consume a diet plan prescribed with LGI recipes prepared with traditional foods of Kerala cuisine as shown in Table 1. This advice was reinforced by the Dietitian throughout the study period.

### 2.5. Follow Up

The subjects were followed up by telephone for verification of dietary compliance; they were evaluated by a 24 h dietary recall at weeks 3, 11, 12, 18, 23 and 24. During the pre-scheduled telephone interviews, the foods consumed by the subject in the preceding 24 h were recalled, and noted. The subjects were asked to come for an in-personal visit to the hospital at week 24 to reassess the anthropometric and biochemical parameters, and the periodically recorded data was statistically analyzed.

### 2.6. Outcomes

Primary outcomes for this study were the effect of LGI diet on HbA1c after 24 weeks of intervention.

Secondary outcomes include changes in blood pressure, lipid profile, fasting insulin, HOMA-IR, ApoB, hs-CRP, body weight and waist circumference after 24 weeks of intervention.

### 2.7. Ethics

This study was approved by the Institutional Ethics Committee of Amrita Institute of Medical Sciences and Research Centre, reference number IEC-AIMS-2018-DIET-165, and registered under Clinical Trials Registry-India (ICMR-NIMS), CTRI Reg No.: CTRI/2019/12/022425.

### 2.8. Statistical Analysis

Descriptive analyses were used to describe the study sample; Chi-square and/or t-test analysis was used to compare the socio-demographic characteristics of the study population. Differences in demographic characteristics were deduced using the χ^2^ test for categorical variables, and nonparametric Wilcoxon test was applied for continuous variables. Paired sample Student’s *t*-test was used to compare the baseline and week 24 within the intervention group and the control group. The independent Student’s t-test was used to compare differences between the intervention and control groups. The mean ± SD difference between the baseline and week 24 for all variables was also calculated. The Pearson correlation coefficient was leaned on to detect associations between the study variables. All statistical analyses were performed using IBM SPSS 20.0 for Windows (SPSS Inc., Chicago, IL, USA). All the statistical tests were based on two-tailed hypothesis tests, where the significance level was defined as *p* < 0.05.

## 3. Results

Table 2 presents a summary of the demographic data of the study participants. In both the intervention and control groups, the mean ages were comparable. Social habits such as smoking, which is a major risk factor for CVD, was not observed in more than 90% of the subjects. Majority of the study participants were engaged primarily in mild physical activity. Comorbidities, such as dyslipidemia and hypertension, were seen in more than 50% of the study subjects. History of CVD and thyroid diseases was seen only in very small fraction of the participants, as shown in Table 2.

Table 3 shows the baseline characteristics of the study variables in both groups. Anthropometric variables and blood glucose profile were comparable at baseline between the groups. Statistically significant difference was observed in LDL between the study groups, all other biochemical variables were comparable at baseline. The nutrient intake showed significant difference in caloric intake between the groups. However the percentage of carbohydrate, protein and fat were comparable.

Table 4 shows the post-intervention changes in all the variables under study. There was a significant reduction in the anthropometric measurements such as weight, BMI and waist circumference. In the LGI group, weight reduced by 1.87 kg and waist circumference reduced by 2.94 cm, whereas not much difference was observed in the control group. The changes observed in the blood glucose profile after intervention were the reduction in fasting and post-prandial blood sugar by 21 mg/dL and 37.87 mg/dL in the LGI diet group, respectively. These changes affected the improvement in HbA1c by 0.93% in the LGI group, which was highly significant compared to the control group. The insulin resistance improved significantly in the intervention group which has shown to reduce the inflammatory markers such as hs-CRP and other cardiovascular risk factors, particularly triglycerides, LDL and ApoB in subjects on LGI diet. The lipid profile and triglycerides showed a highly significant reduction of 26.89 mg/dL in the LGI group compared to an increase in the values in the control group. The total cholesterol and HDL though showed an improvement in the control group were not statistically significant. However, the systolic blood pressure, which is considered an important risk factor toward the prediction of CVD, had shown an insignificant reduction in the intervention group compared to no change in the control group. The diastolic pressure also showed no significant change. Overall, there were significant differences (*p* < 0.05) between the LGI and control groups with respect to weight, glycated hemoglobin, insulin, Homa-IR, triglycerides, hs-CRP and ApoB. In contrast, differences between the two groups were not significant (*p* > 0.05) in relation to systolic and diastolic blood pressure, BMI, waist circumference, fasting blood glucose, postprandial blood glucose, total cholesterol, HDL, LDL and VLDL.

Table 5 shows the comparison of macronutrient intake after 24 weeks of intervention. The protein intake remained unchanged in both groups throughout the study period. There was a significant difference in the percentage of carbohydrate intake and fat intake between the groups.

**Figure 1:**
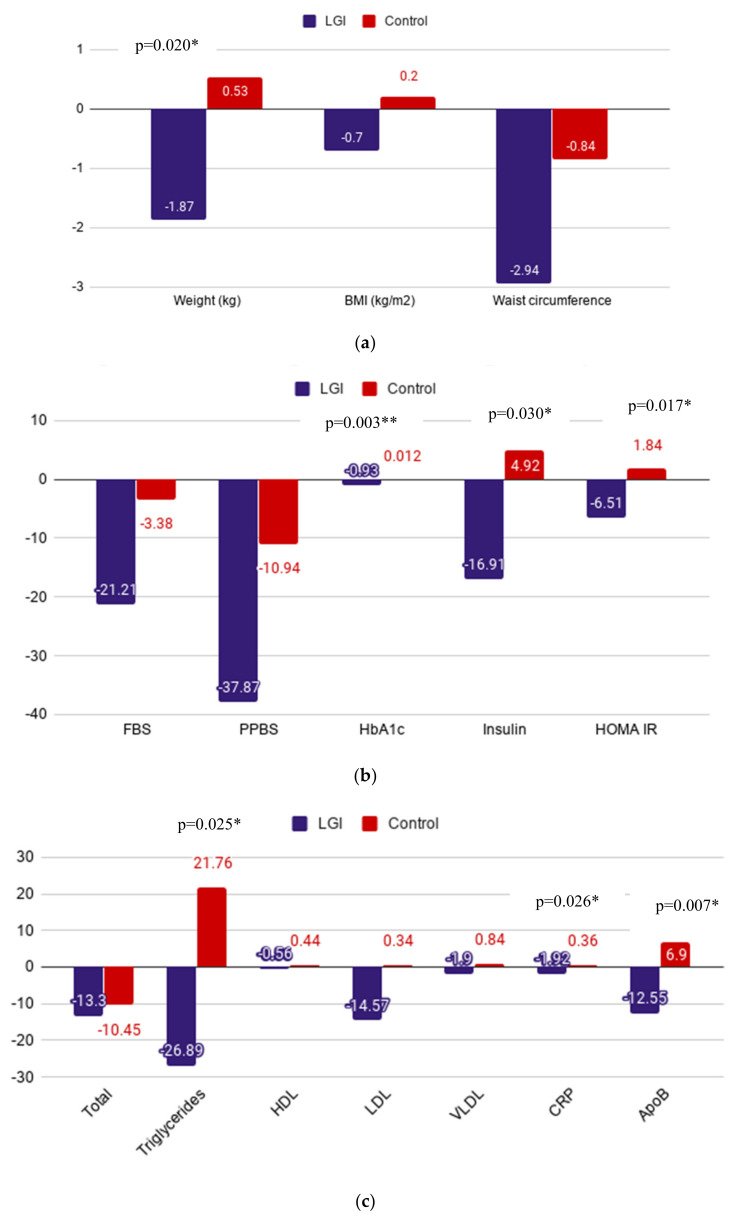
(**a**)—The mean difference in the anthropometric measurements; (**b**)—biochemical variables—blood glucose levels and insulin resistance; and (**c**)—lipid profile and cardiovascular biomarkers after a 24-week diet intervention between the groups. BMI, body mass index; FBS, fasting blood sugar; PPBS, postprandial blood sugar; HbA1c, glycated hemoglobin; HOMA-IR, homeostatic model assessment of insulin resistance; HDL, high density lipoprotein; LDL, low density lipoprotein; VLDL, very low density lipoprotein; hsCRP, high sensitivity C-reactive protein; ApoB, apolipoprotein-B; LGI, low glycemic Index. * Denotes significant difference.

Figure 1a depicts the anthropometric measurements showing a significant weight loss in the LGI group compared to an increase in the control group. There were reductions in fasting and postprandial blood glucose in both intervention and control groups, with a greater reduction observed in the LGI group as shown in Figure 1b. The fasting insulin and insulin resistance, as measured by HOMA-IR shown in Figure 1b, was significantly reduced in LGI group, whereas the same parameter saw an increase in the control group. In contrast to the results obtained in the LGI diet group, there was an increase in all these cardiovascular variables in the control group as presented in Figure 1c.

### Correlation of Weight and Cardiovascular Risk Factors Postintervention in the Study Groups

**Figure 2:**
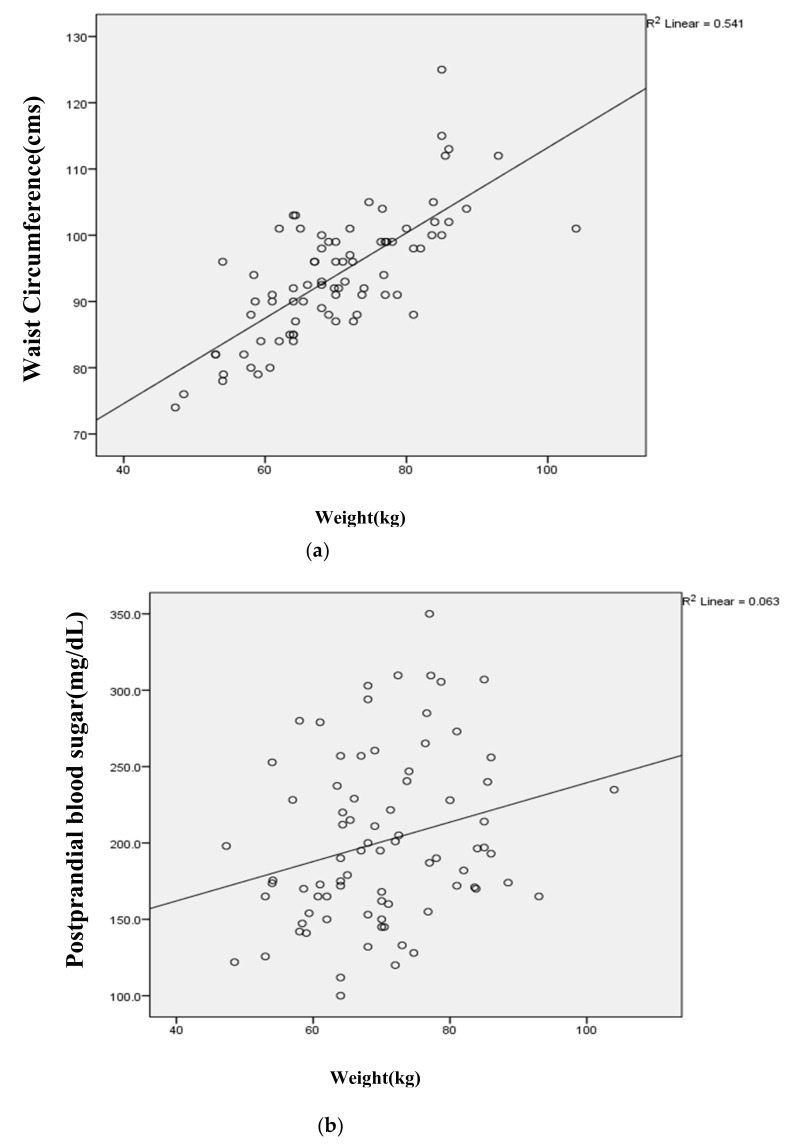

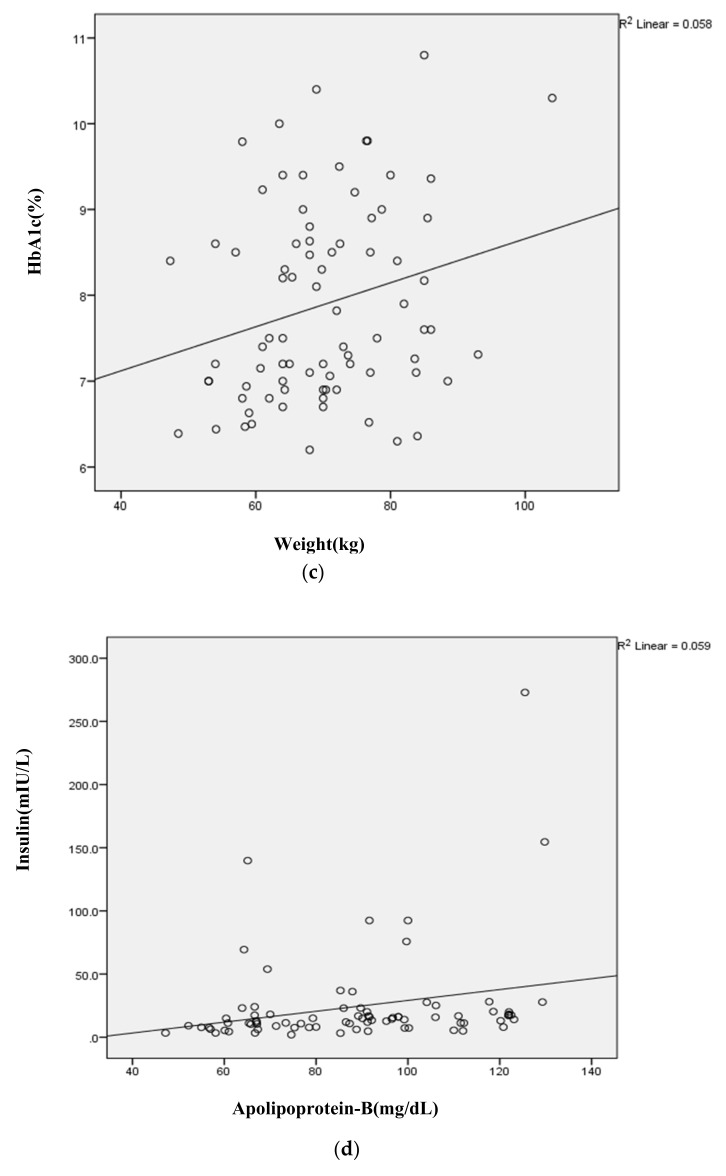
Correlation of weight with anthropometric and biochemical parameters: (**a**) The correlation of weight with WC (waist circumference); (**b**) PPBS (postprandial blood sugar); (**c**) HbA1c after 24 weeks of dietary intervention. (**d**) Correlation of apolipoprotein B with fasting insulin after 24 weeks of dietary intervention.

Association of weight and ApoB with the study variables: Weight was found to be strongly associated with various study variables (Figure 2a–d). A strong positive correlation was observed with waist circumference, whereas a negligible association was found with postprandial blood sugar and HbA1c as depicted in Table 6. Moderate correlations were observed with ApoB and total cholesterol and LDL (Table 7). There was a negligible correlation with fasting insulin levels at the end of the intervention period (Table 7).

## 4. Discussion

The key findings in this randomized controlled trial after 24 weeks intervention with LGI recipes of South Indian Kerala cuisine were significant improvements in glycated hemoglobin and cardiovascular risk factors such as hs-CRP, fasting insulin, HOMA-IR, triglycerides and weight in the intervention group compared with the control group and non-significant reductions in systolic blood pressure, total cholesterol and HDL.

In this intervention study, there was a significant reduction in HbA1c by 0.93% in the LGI group. The UKPDS35 prospective observational study showed that each 1% reduction in HbA1c was associated with a 14% reduction of the relative risk of myocardial infarction [18]. Similarly, the efficacy of a nutritionist-delivered low-GI dietary intervention, with the support of a personal digital assistant (PDA), for adult patients with poorly controlled type II diabetes showed a 0.5% decrease in HbA1c [19]. A study which compared LGI diet with high-cereal fiber diet in type 2 diabetes subjects for a period of 6 months showed a moderately lower HbA1c levels in the intervention group [20]. A 12-week intervention with LGI energy restricted diet in type 2 diabetics did not show any improvement in glycemic control [21]. However, in another study that compared LGI diet and standard diabetic diet, there was no statistically significant difference between the diets in relation to HbA1c and fasting blood glucose [22]. The longer duration of 24 weeks of LGI intervention could be the factor responsible for the better outcome in relation to glycated hemoglobin in this study.

The LGI diet in our study group had a favorable effect on 2-h post prandial blood sugar compared to the control group, albeit the differences were not significant (*p* > 0.05) between the two groups. This is an important finding in T2DM, because high postprandial blood sugar is shown to be a better indicator of CVD risk than fasting blood sugar [23]. Changes in 2-h post prandial blood sugar could be due to changes in insulin sensitivity, which was observed in this study.

This investigation has drawn attention to other metabolic parameters that contribute to the development of CVD risks in patients with type 2 diabetes. For example, fasting insulin and HOMA-IR were noticeably reduced after the 24-week intervention. In contrast to our findings, an intervention with energy restricted LGI diet in 62 overweight women for 6 months did not show any changes in HOMA-IR [24]. Insulin resistance as evaluated by HOMA-IR is considered to be a good predictor for CVD [25]. Elevated risk of CVD observed in insulin resistance is due to the overproduction of reactive oxygen species and advanced glycation end products, which aggravate the low-grade inflammation. Insulin Resistance Atherosclerosis Study (IRAS) showed a direct relation between insulin resistance and atherosclerosis [26].

The present study showed a remarkable difference in serum triglyceride between the low GI and the control groups confirming the findings of a previous study done by Omprakash et al. [27], which reported that a very LGI diet notably abated serum triglycerides in 40 subjects. In contrast, randomized controlled trial performed by Salwa et al. [28] revealed that LGI diet did not show any noteworthy drops in triglyceride levels in type 2 diabetic males. The HDL cholesterol levels, however, remained unchanged in our study. This observation was similar to the findings in other studies [29,30,31]. Hence, the LGI diet of Kerala cuisine is considered safe as the HDL levels did not drop. On the other hand, CRP is a clinical marker of low grade inflammation and this may underlie an increased CV risk known as residual inflammatory index, recently described for patients with type 2 diabetes. Residual Inflammatory Risk (RIR) is defined as persistent circulating levels of hs-CRP > 2mg/L despite an optimal (<70 mg/dL) control of LDL cholesterol and represents an emerging risk factor for the development of cardiovascular events in patients at high risk for atherosclerosis. In our study, the intervention with LGI recipes of Kerala cuisine showed a significant reduction in hs-CRP from 3.3 to 1.4 mg/dL indicating a reduction in RIR, and this reduction can prevent coronary atherosclerosis. A similar observation was seen in the Aggrastat to Zocor (A to Z) trial, which demonstrated that best clinical outcomes occurred when the hsCRP levels were lowered below 2 mg/L in conjunction with lowering LDL-C to < 70 mg/dL [32]. A recent study showed that 39.2% of patients with type 2 diabetes had RIR and the findings suggested that glycemic control, insulin resistance, non-LDL-C lipid variables and central obesity are implicated in RIR in patients with type 2 diabetes even with well-controlled LDL-C levels [33]. These findings support the observations that patients with high inflammatory burden could achieve an hs-CRP <2 mg/L by lifestyle modifications alone [34].

A study done by Jenkins et al. [20] showed that type 2 diabetes subjects who were on high-fiber cereal foods over a duration of 6 months did not show lowered CRP levels. An investigation by Saburo et al. showed high hs-CRP levels in the metabolic diabetic group, significantly higher than non-metabolic diabetic group, indicating the metabolic syndrome subjects who develop type 2 diabetes are at further risk for coronary artery disease (CAD) [1]. The hsCRP measure has been known to have opsonizing properties, increasing the recruitment of monocytes into atheromatous plaque, and inducing endothelial dysfunction by suppression of releases of basal and induced nitric oxide. hsCRP per se has also been found to increase the expression of vascular endothelial plasminogen activator inhibitor-1 (PAI-1) and other adhesion molecules and alter LDL uptake by macrophages [35].

ApoB has been associated with increased coronary artery disease, while LGI diets have been shown to significantly reduce the risk. Findings of this research have affirmed that ApoB was associated with total cholesterol, triglycerides and LDL, but no correlation was observed between ApoB and HDL. Similar observations were recorded by Prabita et al. [36] where ApoB was correlated with total cholesterol, triglycerides, VLDL and LDL. However, HDL was negatively correlated in that study. In the same study, the median serum apolipoprotein B-100 level was significantly higher in diabetes with cardiovascular complications than without complications. In the present study, it was observed that serum ApoB levels had a significant positive correlation with fasting insulin. In a cross-sectional study of 2834 subjects from the Framingham Offspring cohort, GI was shown to be positively associated with the prevalence of the metabolic syndrome and insulin resistance [37]. Insulin resistance, which is a prominent feature of T2DM, may be one of the factors responsible for elevated apolipoprotein B-100 levels in our study subjects. In their study, Jiang’s team [38] confirmed that apo B concentration was significantly higher in diabetic men with cardiovascular complications than in diabetic men without complications.

Our study showed that the LGI preparations of Kerala cuisine had a significant impact on glycemic control and weight loss in the sampled population. It has been proposed that the glycemic index of foods can influence body-weight control [39]. Low-GI foods may also delay the return of hunger by slowing gastric emptying. The LGI preparations of Kerala cuisine, which are high in fiber, promote greater satiety by prolonging the distension of the gastrointestinal tract, causing increased and prolonged secretion of the gut peptides cholecystokinin, ghrelin, glucagons, glucagons-like-peptid-1, and glucose-dependent insulinotropic polypeptide, all of which have been suggested as potential satiety factors compared to other cuisine [40].

This paper, in essence, is the first prospective study, with prolonged follow up, on the effect of LGI Kerala cuisine on glycemic and cardiovascular risk factors. Strict adherence to prescribed diet was ensured by meticulous telephonic follow up. Incorporation of LGI local recipes and traditional ingredients was the highlight of the study that assured unrelented compliance to the diet regimen.

### Limitations of the Study

The dropout rates in the LGI group were high compared to the control group. The reasons put forth, for the withdrawal from the study, were poor compliance to the diet, during social gatherings and travel. Besides, few women, recruited for the study, refused to visit the hospital without a personal escort. 

## 5. Conclusions

The inclusion of LGI diets of Kerala cuisine is an effective strategy of alleviating cardiovascular risk factors in patients with type 2 diabetes. In particular, the long-term implementation of LGI diet of Kerala cuisine has been found to control blood sugar and has been found to promote weight loss, enhance insulin sensitivity and minimize cardiovascular risk.

## Figures and Tables

**Table 1:** The diet plan for the low glycemic index (LGI) diet group.

Breakfast	Red rice puttu (GI = 38) Whole wheat flour (GI = 45) puttu Rolled oats/Steel cut oats puttu (GI = 51/52)
Lunch	Rose Matta rice (GI = 38)
Dinner	Broken wheat (GI = 41) upma Broken wheat + green gram + fenugreek seeds kanji (porridge) Whole wheat flour (GI = 45) roti

**Table 2:** Demographics of the study participants.

	LGI (*n* = 40)	Control (*n* = 40)
Age	54.43 ± 7.57	51.93 ± 7.43
**Gender**		
Male	25 (62.5%)	27 (67.5%)
Female	15 (37.5%)	13 (32.5%)
**Social habits**		
**Smoking**		
Yes	4 (10%)	3 (7.5%)
No	36 (90%)	37 (92.5%)
**Physical activity**		
Sedentary	6 (15%)	4 (10%)
Mild	21 (52.5%)	25 (62.5%)
Moderate	13 (32.5%)	11 (27.5%)
**Comorbidities**		
DLP	22 (55%)	25 (62.5%)
HTN	23 (57.5%)	23 (57.5%)
CAD	11 (27.5%)	6 (15%)
Thyroid	5 (12.5%)	2 (5%)

DLP, dyslipidemia; HTN, hypertension; CAD, coronary artery disease.

**Table 3:** Comparison of the baseline characteristics of the study subjects.

Variable	LGI Group (*n* = 40)	Control Group (*n* = 40)	*p* Value
Blood Pressure			
Systolic BP (mm Hg)	132.9 ± 15.37	131.10 ± 13.28	0.577
Diastolic BP (mm Hg)	79.90 ± 12.32	81.35 ± 8.45	0.541
Anthropometric measurement			
Height (cm)	161.46 ± 7.51	163.98 ± 6.69	0.118
Weight (kg)	69.12 ± 10.78	72.26 ± 10.52	0.191
BMI (kg/m^2^)	26.40 ± 3.03	26.75 ± 3.29	0.623
Waist circumference (cm)	95.34 ± 8.92	96.29 ± 8.99	0.631
Biochemical variables			
FBS (mg/dL)	155.46 ± 36.10	150.01 ± 48.65	0.571
PPBS (mg/dL)	227.21 ± 43.18	223.17 ± 48.53	0.695
HbA1c (%)	8.44 ± 0.96	8.27 ± 0.99	0.443
Insulin (mIU/L)	31.70 ± 46.61	28.65 ± 58.38	0.796
HOMA-IR	11.62 ± 14.63	8.99 ± 13.61	0.408
Total cholesterol (mg/dL)	172.43 ± 34.84	163.42 ± 41.76	0.298
Triglycerides (mg/dL)	147.54 ± 63.20	131.30 ± 42.96	0.183
HDL (mg/dL)	42.48 ± 10.12	41.82 ± 10.48	0.775
LDL (mg/dL)	119.60 ± 32.13	104.50 ± 34.99	0.048 *
VLDL (mg/dL)	27.52 ± 10.75	27.95 ± 12.87	0.872
hsCRP (mg/L)	3.38 ± 3.83	2.79 ± 4.20	0.517
ApoB (mg/dL)	94.44 ± 22.81	88.14 ± 24.61	0.239
Nutrient intake			
Calories	1554.8 ± 233.25	1430.4 ± 182	0.010 **
Carbohydrate (%)	15.71 ± 2.44	15.75 ± 2.09	0.940
Protein (%)	64.62 ± 5.56	63.30 ± 6	0.313
Fat (%)	22.6 ± 5.88	24.3 ± 5.37	0.181
Medications			
Sulfonylureas	24	28	
Metformin	33	42	
DPP4-inh	15	20	
Insulin	13	17	
Acarbose	2	0	
Meglitinides (Repaglinide)	1	0	
Statins	20	26	
Antihypertensives	22	20	

Notes: BP, blood pressure; BMI, body mass index; FBS, fasting blood sugar; PPBS, postprandial blood sugar; HbA1c, glycated hemoglobin; HOMA-IR, homeostatic model assessment of insulin resistance; HDL, high density lipoprotein; LDL, low density lipoprotein; VLDL, very low density lipoprotein; hsCRP, high sensitivity C-reactive protein; ApoB, apolipoprotein-B. * (*p* < 0.05); ** (*p* < 0.01)

**Table 4:** Comparison of mean ± SD of the variables at baseline and 24 weeks after intervention between groups.

Variable	Group	Baseline	24 Weeks	Mean Difference	*p* Value	*p* Value (LGI vs. Control) at 24 Weeks
Blood Pressure						
Systolic pressure (mmHg)	LGI	132.90 ± 15.32	130.35 ± 17.63	−2.55 ± 16.6	0.337	0.683
	Control	131.1 ± 13.28	131.83 ± 14.43	0.72 ± 14.84	0.759	
Diastolic pressure (mmHg)	LGI	79.9 ± 12.32	77.63 ± 7.08	−2.27 ± 12.67	0.263	0.068
	Control	81.35 ± 8.45	80.60 ± 7.29	−0.75 ± 6.96	0.500	
Anthropometric measurements						
Weight (kg)	LGI	69.12 ± 10.78	67.25 ± 9.81	−1.87 ± 2.63	0.000 **	0.020 *
	Control	72.26 ± 10.52	72.79 ± 11.00	0.53 ± 1.96	0.094	
BMI (kg/m^2^)	LGI	26.40 ± 3.03	25.71 ± 2.77	−0.70 ± 0.99	0.000 **	0.078
	Control	26.75 ± 3.29	26.95 ± 3.42	0.20 ± 0.70	0.083	
Waist circumference (cm)	LGI	95.34 ± 8.92	92.40 ± 8.99	−2.94 ± 4.71	0.000 **	0.148
	Control	96.29 ± 8.99	95.45 ± 9.64	−0.84 ± 4.47	0.244	
Biochemical parameters						
FBS (mg/dL)	LGI	155.46 ± 36.10	136.75 ± 27.54	−21.21 ± 39.42	0.001 **	0.192
	Control	150.01 ± 48.65	146.63 ± 38.73	−3.38 ± 34.57	0.540	
PPBS (mg/dL)	LGI	227.21 ± 43.18	189.33 ± 56.49	−37.87 ± 59.36	0.000 **	0.063
	Control	223.17 ± 48.53	212.22 ± 52.13	−10.94 ± 51.44	0.186	
HbA1c (%)	LGI	8.44 ± 0.96	7.52 ± 0.94	−0.93 ± 0.92	0.000 **	0.003 **
	Control	8.27 ± 0.99	8.27 ± 1.20	0.012 ± 0.87	0.929	
Insulin (mIU/L)	LGI	31.70 ± 46.61	14.79 ± 15.89	−16.91 ± 40.42	0.012 *	0.030 *
	Control	28.65 ± 58.38	33.57 ± 51.17	4.92 ± 57.67	0.592	
HOMA-IR	LGI	11.62 ± 14.63	5.10 ± 6.06	−6.51 ± 12.11	0.002 **	0.017 *
	Control	8.99 ± 13.61	10.83 ± 13.50	1.84 ± 15.76	0.464	
Total Cholesterol (mg/dL)	LGI	172.43 ± 34.84	159.12 ± 33.68	−13.30 ± 37.37	0.030 *	0.422
	Control	163.42 ± 41.76	152.97 ± 34.41	−10.45 ± 31.77	0.044 *	
Triglycerides (mg/dL)	LGI	147.54 ± 63.20	120.65 ± 44.80	−26.89 ± 48.37	0.001 **	0.025 *
	Control	131.30 ± 42.96	153.06 ± 77.60	21.76 ± 56.69	0.020 *	
HDL (mg/dL)	LGI	42.48 ± 10.12	41.92 ± 11.22	−0.56 ± 5.64	0.531	0.886
	Control	41.82 ± 10.48	42.27 ± 10.50	0.44 ± 6.86	0.683	
LDL (mg/dL)	LGI	119.60 ± 32.13	105.02 ± 26.80	−14.57 ± 27.42	0.002 **	0.979
	Control	104.50 ± 34.99	104.85 ± 33.13	0.34 ± 28.44	0.939	
VLDL (mg/dL)	LGI	27.52 ± 10.75	25.62 ± 11.24	−1.90 ± 10.28	0.249	0.254
	Control	27.95 ± 12.87	28.79 ± 13.34	0.84 ± 9.71	0.588	
hs-CRP (mg/L)	LGI	3.38 ± 3.83	1.46 ± 1.04	−1.92 ± 3.83	0.003 **	0.026 *
	Control	2.79 ± 4.20	3.16 ± 4.61	0.36 ± 3.21	0.479	
ApoB (mg/dL)	LGI	94.44 ± 22.81	81.88 ± 19.83	−12.55 ± 19.50	0.000 **	0.007 **
	Control	88.14 ± 24.61	95.05 ± 22.27	6.90 ± 16.15	0.010 **	
Nutrient intake						
Calories (kcal)	LGI	1430.4 ± 182	1511 ± 137.5	80.6 ± 243	0.043 *	0.010 **
	Control	1554.8 ± 233.3	1450.4 ± 157.1	104.4 ± 250.1	0.012	
Protein (g)	LGI	55.8 ± 8.49	61.8 ± 10.1	6.02 ± 14.95	0.015 *	0.025 *
	Control	61.3 ± 12.8	58.5 ± 11.7	−2.88 ± 8.15	0.221	
Carbohydrate (g)	LGI	230.9 ± 33.9	232.7 ± 28.4	1.85 ± 47.11	0.807	0.081
	Control	245.1 ± 37.5	238.4 ± 26.4	−6.69 ± 46.2	0.365	
Fat (g)	LGI	36.1 ± 10.4	40.9 ± 7.71	1.77 ± 7.47	0.036 *	0.019 *
	Control	42.4 ± 12.7	34.2 ± 10.1	−8.15 ± 17.2	0.005 **	

BMI, body mass index; FBS, fasting blood sugar; PPBS, postprandial blood sugar; HbA1c, glycated hemoglobin; HOMA-IR, homeostatic model assessment of insulin resistance; HDL, high density lipoprotein; LDL, low density lipoprotein; VLDL, very low density lipoprotein; hsCRP, high sensitivity C-reactive protein; ApoB, apolipoprotein-B. * (*p* < 0.05); ** (*p* < 0.01)

**Table 5:** Comparison of the percentage of the macronutrient intake between baseline and 24 weeks after dietary intervention in the study groups.

Nutrient Intake		Baseline	24 Weeks	*p* Value
Calories (kcal)	LGI	1430.4 ± 182	1511 ± 137.5	0.07
	Control	1554.8 ± 233.3	1450.4 ± 157.1	
Protein (%)	LGI	15.71 ± 2.44	16.38 ± 2.44	0.571
	Control	15.75 ± 2.09	16.07 ± 2.53	
Carbohydrate (%)	LGI	64.62 ± 5.56	61.6 ± 4.62	<0.001 **
	Control	63.3 ± 6	65.93 ± 5.14	
Fat (%)	LGI	22.6 ± 5.88	24.37 ± 3.9	0.003 **
	Control	24.3 ± 5.37	21.12 ± 5.4	

** (*p* < 0.01)

**Table 6:** Correlation between weight and other study variables after 24 weeks of diet intervention.

Table 4 Variables	Weight		*p* Value
	Pearson Correlation Coefficient (r)		
Waist circumference	0.735 **		<0.01
Postprandial blood sugar	0.251 *		<0.05
HbA1c	0.241 *		<0.05

* (*p* < 0.05); ** (*p* < 0.01).

**Table 7:** Correlation between apolipoprotein-B and other study variables after 24 weeks of diet intervention.

Variables	ApoB	*p* Value
	Pearson Correlation Coefficient (r)	
Total Cholesterol	0.529 **	<0.01
Triglycerides	0.314 **	<0.01
LDL	0.599 **	<0.01
Fasting Insulin	0.237 *	<0.05

* (*p* < 0.05); ** (*p* < 0.01).
